# The response of seed germination and seedling growth of *Chamerion angustifolium* to drought stress

**DOI:** 10.3389/fpls.2026.1802920

**Published:** 2026-03-31

**Authors:** Xiaojuan Liu, Caiwei Zhang

**Affiliations:** College of Forestry, Gansu Agricultural University, Lanzhou, China

**Keywords:** *Chamerion angustifolium*, drought resistance, physiological characters, seed germination, seedling growth

## Abstract

**Introduction:**

*Chamerion angustifolium* (L.) Holub is a species with potential ecological and horticultural value, yet its tolerance to drought stress during early life stages remains poorly understood. This study investigated the drought tolerance of *C. angustifolium* by evaluating its physiological responses at both seed germination and seedling growth stages.

**Methods:**

Seed germination assays were conducted under osmotic stress induced by polyethylene glycol (PEG-6000) at six specific concentrations: 2%, 4%, 6%, 8%, 10%, and 12% (w/v), with ultrapure water serving as the control. Germination parameters, including germination rate, vigor, index, relative drought damage rate, and post-germination radicle and plumule lengths, were recorded. Additionally, the contents of soluble sugars and soluble proteins in the seeds were quantified. For the seedling experiments, drought stress was then initiated by precisely controlling and maintaining the soil water content (SWC) at three deficit levels: 30% ± 5%, 50% ± 5%, and 70% ± 5%, representing mild, moderate, and severe stress, respectively. Each stress level was applied for 5, 10, or 15 days, corresponding to short-, medium-, and long-term exposure. Seedlings maintained at 90% ± 5% SWC throughout the experiment served as the wellwatered control.

**Results:**

(1) Under PEG-6000 solutions of varying concentrations and different stress durations, seed germination rate, relative germination rate, germination vigor, germination index, and the radicle andplumule growth of *C. angustifolium* were significantly lower than the control (P<0.05). Seeds failed to germinate at all under 12% concentration; As solution concentration increased and stress duration prolonged, both radicle and plumule lengths exhibited a decreasing trend after seedling emergence; Under PEG-6000 solutions of varying concentrations, both soluble sugar and soluble protein contents in *C. angustifolium* seeds were significantly higher than the control level (P<0.05). As stress duration increased, soluble sugar and soluble protein contents generally showed an upward trend across all PEG-6000 concentrations.(2) Under different drought stress treatments, the osmotic regulator content and antioxidant enzyme activity in *C. angustifolium* seedlings were significantly higher (P< 0.05) than in the control group. With prolonged stress duration, osmotic regulator content showed an upward trend, while antioxidant enzyme activity exhibited a downward trend.

**Conclusion:**

In summary, *C. angustifolium* possesses a certain degree of drought tolerance, and short-term mild drought stress does not significantly affect seed germination or seedling growth.

## 1 Introduction

Drought stress represents a major environmental constraint limiting plant growth, distribution, and survival, particularly in arid and semi-arid regions (Qi et al., 2011). At the cellular and physiological levels, water deficit disrupts osmotic balance, inhibits photosynthesis, induces oxidative damage through reactive oxygen species (ROS) accumulation, and impairs nutrient uptake (Shanker et al., 2014). In response, plants have evolved a suite of adaptive strategies, including the accumulation of osmoprotectants (e.g., proline, soluble sugars and proteins) for osmotic adjustment, and the upregulation of antioxidant enzymes (e.g., superoxide dismutase, peroxidase, catalase) to scavenge ROS and maintain cellular homeostasis (Nasir and Toth, 2022). These responses are crucial across different life stages, with seed germination and seedling establishment being exceptionally vulnerable. Drought can severely reduce seed germination percentage and vigor by limiting water imbibition and radicle emergence, while in seedlings, it often restricts root and shoot growth, reduces biomass, and triggers complex physiological reprogramming to enhance stress acclimation (Makbul et al., 2011). Despite these general responses being well-characterized in many species, they are often species-specific, necessitating individual evaluation for plants of horticultural interest.

*Chamerion angustifolium* (L.) Holub, a perennial herb widely distributed across northern temperate zones including Northwest China (Wu and Hong, 2001), has attracted increasing attention for its ornamental value and ecological adaptability (Quan et al., 2010). Its well-formed plant architecture, prolonged flowering period, and pronounced stress resilience make it a promising candidate for landscape use in challenging environments. Previous research has largely focused on the species’ introduction, domestication, and general horticultural traits (Zhai et al., 2009; Quan et al., 2011; Lin et al., 2018; Xv, 2003; Lv, 2004; He and He, 2011; Lei, 2003) Recent studies have begun to reveal the drought stress response mechanisms of *C. angustifolium*. For instance, seedlings have been shown to activate antioxidant enzymes (superoxide dismutase, peroxidase, and catalase) under drought conditions, with seedling growth significantly inhibited when soil moisture content falls below 60% (Xyu, 2023). Additionally, under PEG-simulated drought stress, seed germination and seedling physiological parameters, including antioxidant enzyme activities, are significantly affected, with germination completely inhibited at PEG concentrations above 15% (Ye et al., 2024). These findings indicate that *C. angustifolium* exhibits measurable physiological responses to drought stress, though its tolerance is limited under severe conditions. Additionally, studies on its subspecies *C. angustifolium* subsp. *circumvagum* indicate that this species exhibits a certain degree of drought tolerance. However, under severe drought conditions, it experiences impaired photosynthetic function and complex changes in physiological parameters (Zhang, 2024). Another subspecies, *C. angustifolium* subsp. *angustifolium*, is widely recognized for its role as a pioneer species in post-fire ecosystems (Liu et al., 2022).

Therefore, this study aims to: (1) evaluate the effects of simulated drought stress (using PEG-6000) on seed germination parameters and early radicle/plumule growth in *C. angustifolium*; (2) investigate the physiological responses of its seedlings to controlled soil water deficit, focusing on changes in osmotic regulators and antioxidant enzyme activities; and (3) identify the stress thresholds and acclimation strategies employed during these early developmental stages. The findings will elucidate the species’ drought tolerance capacity and provide a scientific basis for its selection and utilization in greening projects across arid and semi-arid regions, such as northwest China.

## 2 Methods

### 2.1 Experimental material

#### 2.1.1 Seed source and pretreatment

Seeds of *Chamerion angustifolium* were collected from Mapo Township, Yuzhong County, Lanzhou City, Gansu Province, and were subsequently sealed and stored dry at room temperature until use. Before sowing, the seeds were surface-sterilized by soaking in a 20% NaClO solution for 1.5 min, followed by rinsing with distilled water 5–6 times. They were then immersed in water at 40 °C for 12 h to promote germination. This temperature was selected based on established protocols for *C. angustifolium* seed treatment to soften the seed coat and enhance imbibition (Du and Chang, 2011; Lyu et al., 2009).

#### 2.1.2 Substrate composition and properties

The *C. angustifolium* seedling substrate used was a commercial potting mix, “Strong Seedling No.1” (Gansu Luneng Agricultural Science and Technology Co. Ltd., China). The substrate exhibited a maximum field water holding capacity of 75.52%. Key physicochemical properties of the substrate were as follows: pH 6.3, total carbon 85.734 mg·g^-^¹, total nitrogen 6.736 mg·g^-^¹, total phosphorus 0.797 mg·kg^-^¹, bulk density of 1.537 g·cm^-^³, and water content of 0% (measured by the ring knife method; Physics Laboratory of Nanjing Institute of Soil Science, Chinese Academy of Sciences, 1978). Prior to use, the substrate was air-dried.

#### 2.1.3 Seedling cultivation and experimental setup

The prepared substrate was packed into pots (15.8 cm in diameter and 13.1 cm in height), with each pot filled with a standardized amount of 400 g. Three holes were made in each pot with 10 seeds per hole at a depth of 0.5 cm. For the seedling experiment, each treatment included three independent biological replicates. Each biological replicate consisted of three pots as technical replicates, resulting in a total of nine pots per treatment. After emergence, six seedlings with uniform growth were retained in each pot. Drought stress treatment was initiated one month after sowing. Drought stress treatment was initiated one month after sowing. At this stage, the seedlings had developed to a uniform growth phase. Morphological measurements taken prior to treatment initiation showed that the plants had an average height of 15.23 ± 0.5 cm, a basal diameter of 3.2 ± 0.3 mm. This uniformity ensured a consistent physiological baseline for all subsequent drought stress treatments.

### 2.2 Experiment design

#### 2.2.1 The effect of PEG-6000 solution simulating drought stress on the germination of *C. angustifolium* seeds

This study employed PEG-6000 solutions to simulate drought stress environments of varying intensities. During the experimental material preparation phase, fully developed, healthy, disease-free seeds of *C. angustifolium* were selected as the research subjects. The specific experimental design is as follows: Using ultrapure water as the blank control (CK), six drought stress gradients were established with PEG-6000 solution concentrations of 2%, 4%, 6%, 8%, 10%, and 12% (w/v). The corresponding water potentials (ψs) of these solutions at 25 °C were calculated according to the formula of Michel and Kaufmann (1973), yielding values of approximately -0.05, -0.10, -0.20, -0.35, -0.55, and -0.80 MPa, respectively.

Each concentration treatment included seven independent biological replicates, with 50 seeds per replicate. Only visually healthy seeds were used. The selection criteria included: an intact seed coat, uniform size and fullness, and the absence of any visible symptoms of fungal infection (e.g., mycelia, discoloration) or insect damage. Additionally, after surface sterilization (as described in section 2.1.1), any seeds that floated or appeared softened were also discarded, as these characteristics typically indicate non-viable or deteriorated embryos (International Seed Testing Association (ISTA), 2022), to ensure only viable, disease-free material was used in the germination assays.

They were placed in germination trays lined with filter paper saturated with PEG-6000, maintaining adequate spacing between seeds to guarantee sufficient oxygen during germination. Following these preparatory steps, the simulated drought stress germination experiment commenced and lasted for 7 days. This duration was determined based on the germination kinetics of *C. angustifolium* reported in recent literature. Ye et al. (2024) observed seed germination daily until no new germination occurred for five consecutive days, and their data showed that the majority of germination events for this species were completed within the first 7 days under both control and PEG-stressed conditions. Therefore, a 7-day period was considered sufficient to capture the peak germination response while minimizing confounding effects from prolonged PEG exposure, consistent with general guidelines for seed testing in herbaceous species (International Seed Testing Association (ISTA), 2022).

During this period, water was replenished to each treatment bed every 24 hours using the weighing method to restore it to its initial weight, thereby maintaining a constant water potential. Throughout the germination process, the temperature was controlled at 25 °C, humidity at 70%, and light intensity at 5000 lx. Germination observations commenced on the day of seed inoculation. Germination was defined as the radicle protruding through the seed coat to a length equal to or exceeding the seed diameter. If no germination signs appeared within a 3-day observation period, the batch was deemed non-germinating. During the germination phase, observations were conducted at fixed times daily, with data recorded. Among the seven biological replicates, three were used for non-destructive monitoring of germination parameters (germination rate, vigor, index, and radicle/plumule growth). The remaining four biological replicates were used for destructive sampling to measure soluble sugar and protein content at 1,3,5, and 7 days post-germination. The germination percentage (Gp) was calculated on the 7th day post-germination.

#### 2.2.2 The effects of drought stress on the physiological characteristics of *C. angustifolium* seedlings

The experimental factors were drought stress intensity and drought stress duration; Stress intensity was set at four levels: mild (T1), moderate (T2), severe (T3), and normal control (CK). The corresponding soil relative water contents for these gradients were designed according to the plant water gradient classification proposed by Hsiao (1973), corresponding to 70% ± 5%, 50% ± 5%, 30% ± 5%, and 90% ± 5% of field capacity, respectively; Drought stress duration comprised short-term (5 days), medium-term (10 days), and long-term (15 days) exposure. Each treatment combination (stress level × duration) included three independent biological replicates, with each biological replicate consisting of three pots as technical replicates (as described in section 2.1.3).

Based on the set relative soil moisture content and dry weight of the potting soil, calculate the target weight per pot after watering for each treatment. This study employed precise water control methods for experimental materials. Based on the preset moisture gradient range, supplemental irrigation was initiated when soil moisture content dropped to the lower threshold, continuing until the upper moisture limit was reached. Irrigation volume was measured through periodic weighing, with containers weighed every 48 hours and each watering volume meticulously recorded. After 15 days, the controlled experiment concluded. Six plants were randomly selected from each treatment replicate. Leaf samples (the second to fourth true leaves counted from the top) were collected on days 5, 10, and 15 post-stress to measure physiological indicators.

### 2.3 Index determination

#### 2.3.1 Determination of seed indicators

Seed-related germination indices were determined according to the method of Cardarelli et al. (2022) (see **Table 1**).

**Table 1:** Calculation formula for relevant germination indicators.

Germination indicators(GI)	Computing formula
Germination rate	The cumulative number of germinated seeds on the 7th day/the number of seeds tested×100%
Relative germination rate	Stress treatment germination rate/control treatment germination rate×100%
Germinative force	The peak germination number at the early stage of seed germination/the number of tested seeds×100%
Germinative index	∑Gt/Dt(Gt represents the germination value increase on the TTH day, and Dt represents the corresponding number of days)
Relative drought damage rate	(Control treatment germination rate - stress treatment germination rate)/Control treatment germination rate×100%

Measure the length of the radicle and plumule after seed germination using the scientific image processing software ImageJ. Growth increment = measurement on day (n) - measurement on day (n-1).

The anthrone colorimetric method was employed to determine the soluble sugar content in *C. angustifolium* seeds (Samarah et al., 2009); the Coomassie Brilliant Blue method was used to measure the soluble protein content in *C. angustifolium* seeds (Yu et al., 2016).

#### 2.3.2 Physio-biochemical indices

Malondialdehyde (MDA) Content: The MDA content, indicative of lipid peroxidation, was determined using the thiobarbituric acid (TBA) reaction method as described by Hodges et al. (1999).

Osmoregulatory Substances: Soluble Sugar (Ss) Content was determined using the anthrone-sulfuric acid method following the protocol of Zhang et al. (2006). Soluble Protein (Sp) Content was assayed by the Coomassie Brilliant Blue G-250 staining method, using bovine serum albumin as a standard, as outlined by Bradford (1976).

Proline (Pro) Content was measured by the acid-ninhydrin method following extraction with 3% sulfosalicylic acid, based on the procedure of Bates et al. (1973).

Antioxidant Enzyme Activities: Frozen leaf tissue was homogenized in an ice-cold 50 mM phosphate buffer (pH 7.8) containing 1% (w/v) polyvinylpyrrolidone. The homogenate was centrifuged at 12,000 × g for 20 min at 4 °C, and the resultant supernatant was used as the crude enzyme extract for the following assays (Madamanchi et al., 1994).

Superoxide Dismutase (SOD) activity was determined by measuring its capacity to inhibit the photochemical reduction of nitroblue tetrazolium (NBT) at 560 nm, following the methodology of Kamran et al. (2021).

Peroxidase (POD) activity was assayed by monitoring the oxidation of guaiacol at 470 nm, as described by Kamran et al. (2021).

Catalase (CAT) activity was measured by tracking the decomposition of H_2_O_2_ at 240 nm, according to the method of Kamran et al. (2021).

The relative water content (RWC) of *C. angustifolium* leaves was determined by the drying and weighing method (Türk and Erdal, 2015). After weighing 3 g of *C. angustifolium* seedling leaves from each experimental treatment group, the leaves were wiped clean and weighed as fresh weight (*Wf*). After weighing, the leaf materials were immersed in ultrapure water for 4 h. At this time, the *C. angustifolium* leaves were saturated with water, and after removing the leaves, the surface water was sucked dry, and the weight of the *C. angustifolium* leaves at this time was weighed as saturated weight (*Wt*), then pack the weighed, saturated leaves into a dry envelope. Then put the saturated weight of *C. angustifolium* leaves in a dry envelope, put it in a 100-105 °C wind drying oven it for 15 min, then lower the temperature of the wind drying oven to 85 °C and bake it to a constant weight, then take out the envelope, and when the envelope cooled down to the room temperature, take out the dried *C. angustifolium* leaves, and then weigh the dry weight of the *C. angustifolium* leaves (*Wd*). Reference formula for the calculation of relative water content:


$$RWC=\frac{Wf-Wd}{Wt-Wd}\times 100\%$$

### 2.4 Data analysis

Microsoft Excel 2010 for data organization, and data analysis was carried out using SPSS 26.1 software. The significance of differences between treatments was tested using Duncan’s multiple range test. Correlation analysis and principal component analysis were also performed; Graphs were created using Origin 2021 software from two independent experiments.

## 3 Results

### 3.1 Effect of PEG-6000 solution simulated drought stress on the relevant indicators of *Chamerion angustifolium* seeds

The effects of PEG-6000 solutions at different concentrations on germination-related indices of *Chamerion angustifolium* seeds are shown in **Table 2**. Under treatments with PEG-6000 solutions at various concentrations, germination rate, relative germination rate, germination vigor, and germination index were all significantly lower than the control (*P*< 0.05). As PEG-6000 concentration increased, seed germination parameters generally exhibited an overall downward trend compared to the control. However, a biphasic response was observed: at the low concentration of 4%, the germination rate (54.00%) was significantly higher than that at 2% (41.33%) (*P<*0.05, **Table 2**), suggesting a potential hormetic effect where mild osmotic stress slightly stimulated germination. As concentration continued to increase beyond 6%, germination parameters declined sharply. The most pronounced decrease in germination rate occurred at 8% and 10% concentrations, with almost complete inhibition at 12% (**Table 2**). The relative drought damage rate of *C. angustifolium* seeds generally increased with rising solution concentration. At 12% concentration, the relative drought damage rate reached 100%, with complete seed failure to germinate.

**Table 2:** Relevant germination indicators of *Chamerion angustifolium* seeds under different concentrations of PEG-6000.

PEG-6000 concentration	Germination rate%	Relative germination rate%	Germinative force%	Germinative index	Relative drought damage rate%
0%	81.33 ± 1.33a	/	66.00 ± 4.44a	18.76 ± 0.91a	/
2%	41.33 ± 2.67c	50.79 ± 2.99b	36.00 ± 3.27bc	11.09 ± 0.95bc	49.21 ± 1.33c
4%	54.00 ± 1.16b	66.39 ± 0.73a	41.00 ± 3.30b	12.35 ± 0.33b	33.61 ± 2.67d
6%	40.00 ± 2.33c	49.05 ± 4.05b	28.67 ± 2.86c	9.54 ± 0.20c	50.95 ± 1.16c
8%	10.67 ± 2.67d	13.17 ± 3.42c	6.67 ± 1.23d	3.10 ± 0.92d	86.83 ± 4.00b
10%	1.33 ± 1.33e	1.67 ± 1.67d	1.33 ± 0.84d	0.44 ± 0.01de	98.33 ± 2.67a
12%	/	/	/	/	100.00 ± 1.33a

Different lowercase letters indicate that the data of each treatment group is significantly different at the 0.05 level.

Values in parentheses indicate the corresponding water potential (ψ_s_​, MPa) calculated at 25 °C according to Michel and Kaufmann (1973).

### 3.2 Effects of PEG-6000 solution simulating drought stress on post-germination radicle length and plumule growth of *C. angustifolium* seedlings

**Figure 1:**
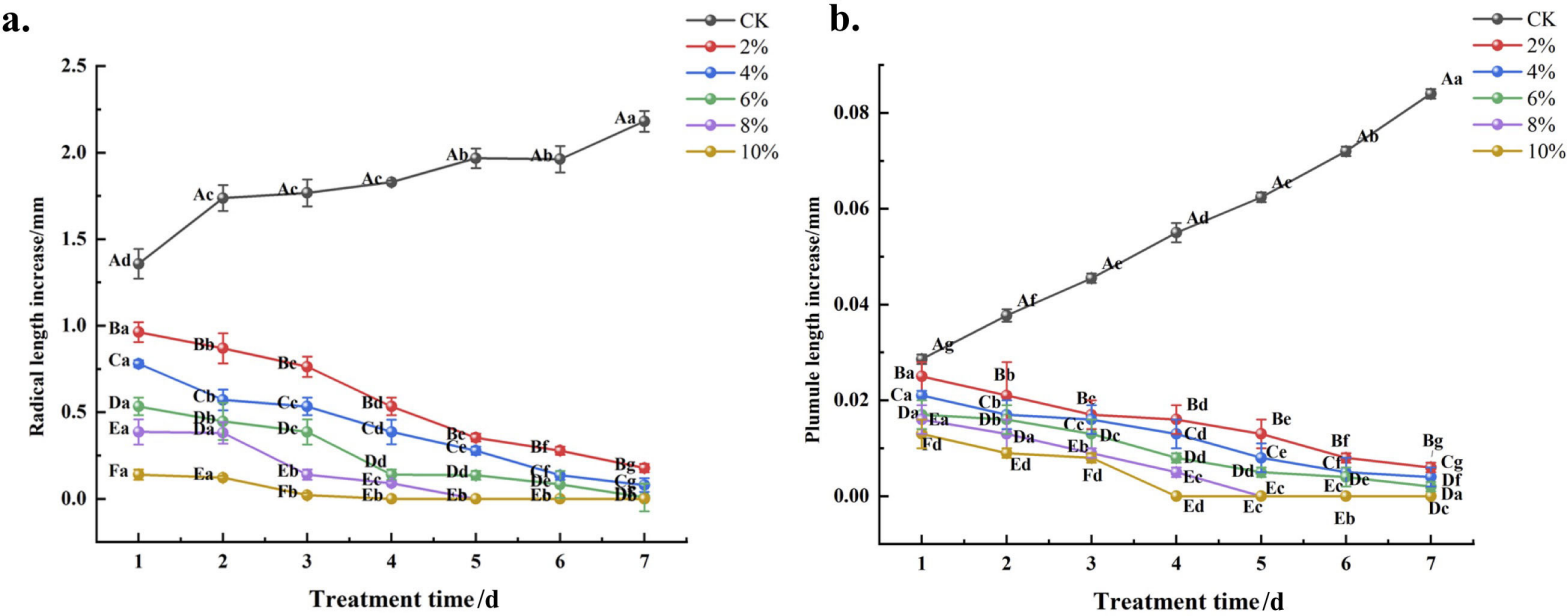
Effects of different concentrations of PEG-6000 on the daily growth increments of radicle length and plumule length of *Chamerion angustifolium* seedlings after germination **(A)** the daily growth increments of radicle length; **(B)** the daily growth increments of plumule length. Growth increment = length on day n − length on day n-1. Different lowercase letters indicate significant difference between the same drought treatment and different stress times at the 0.05 level; Different capital letters indicate that there is significant difference between different drought treatments under the same stress time at the 0.05 level, the same as below.

The effects of different concentrations of PEG-6000 solution on the daily growth increments of radicle and plumule length in germinated *C. angustifolium* seeds are shown in **Figure 1**. As defined in section 2.3.1, the growth increment (length on day n minus length on day n-1) reflects the growth rate of the seedlings. Under the same stress duration, both radicle and plumule length increments decreased with increasing solution concentration. As stress duration extended, the growth increments gradually decreased in all PEG treatments, indicating a slowing of growth rate under prolonged stress. In contrast, the control (CK) seedlings maintained relatively stable growth increments throughout the observation period, showing an overall increase in cumulative length.

### 3.3 Effects of PEG-6000 solution simulating drought stress on seed soluble sugars and protein content

**Figure 2:**
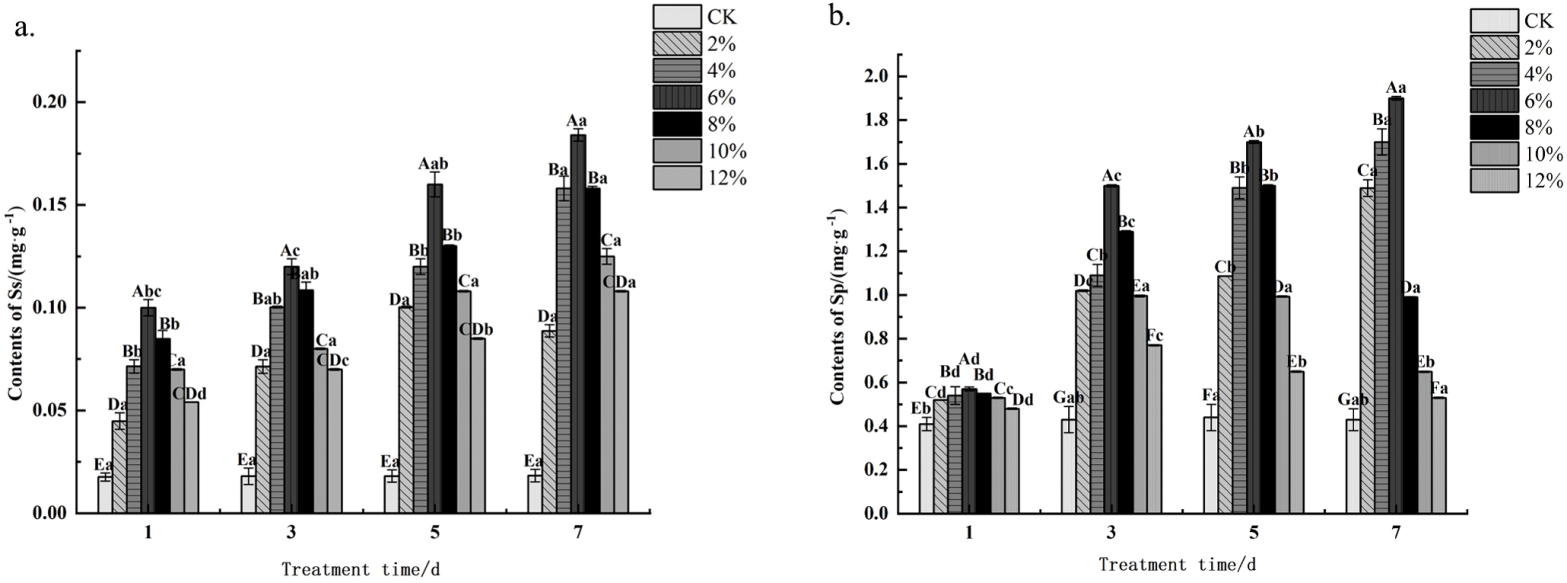
Effects of PEG-6000 solution simulates drought stress at different concentrations on contents of permeation-regulating substance of *Chamerion angustifolium* seeds **(A)** contents of soluble sugar; **(B)** contents of soluble protein.

Measurements of soluble sugar and soluble protein content revealed (**Figure 2**) that under all concentration treatments, soluble sugar and soluble protein levels were significantly higher (*P*< 0.05) than the control level at different stress time points. At different stress durations, the 6% concentration treatment exhibited the highest soluble sugar content, with the peak value observed at day 7 (0.184 mg·g^-1^). The 6% concentration treatment also showed the highest soluble protein content across all stress durations, reaching a maximum of 1.9 mg·g^-1^ at day 7. At stress durations of 1d, 3d, and 5d, the 6% concentration treatment also showed the highest soluble protein content. As stress duration increased, soluble sugar content in *C. angustifolium* seeds treated with all concentrations showed an increasing trend. Soluble protein content in seeds treated with 2%, 4%, and 6% concentrations also exhibited an increasing trend. In contrast, soluble protein content in seeds treated with 8%, 10%, and 12% concentrations first increased and then decreased.

### 3.4 Effect of drought stress on the relative water content of leaves of *C. angustifolium* seedlings

**Figure 3:**
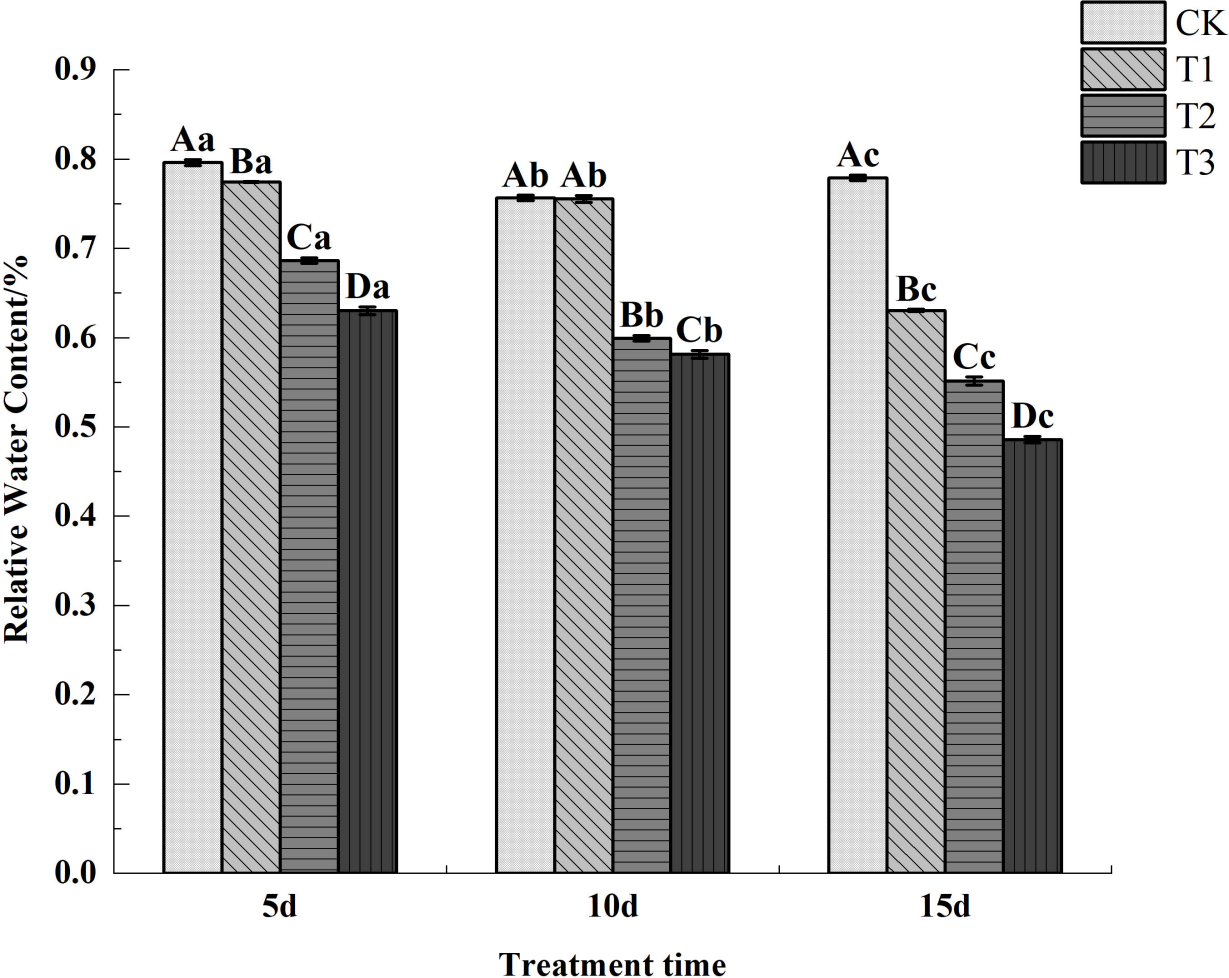
Changes of relative water content in Chamerion angustifolium seedlings leaves under drought stress.

Measurements of RWC revealed that all treatments exhibited significantly lower RWC compared to the control. Among the treatments, leaf relative water content consistently followed the pattern T1 > T2 >T3, with highly significant differences between treatments. Within each treatment, leaf relative water content decreased progressively as stress duration increased (**Figure 3**).

### 3.5 Effects of drought stress on MDA and osmoregulatory substance content in leaves of *C. angustifolium* seedlings

**Figure 4:**
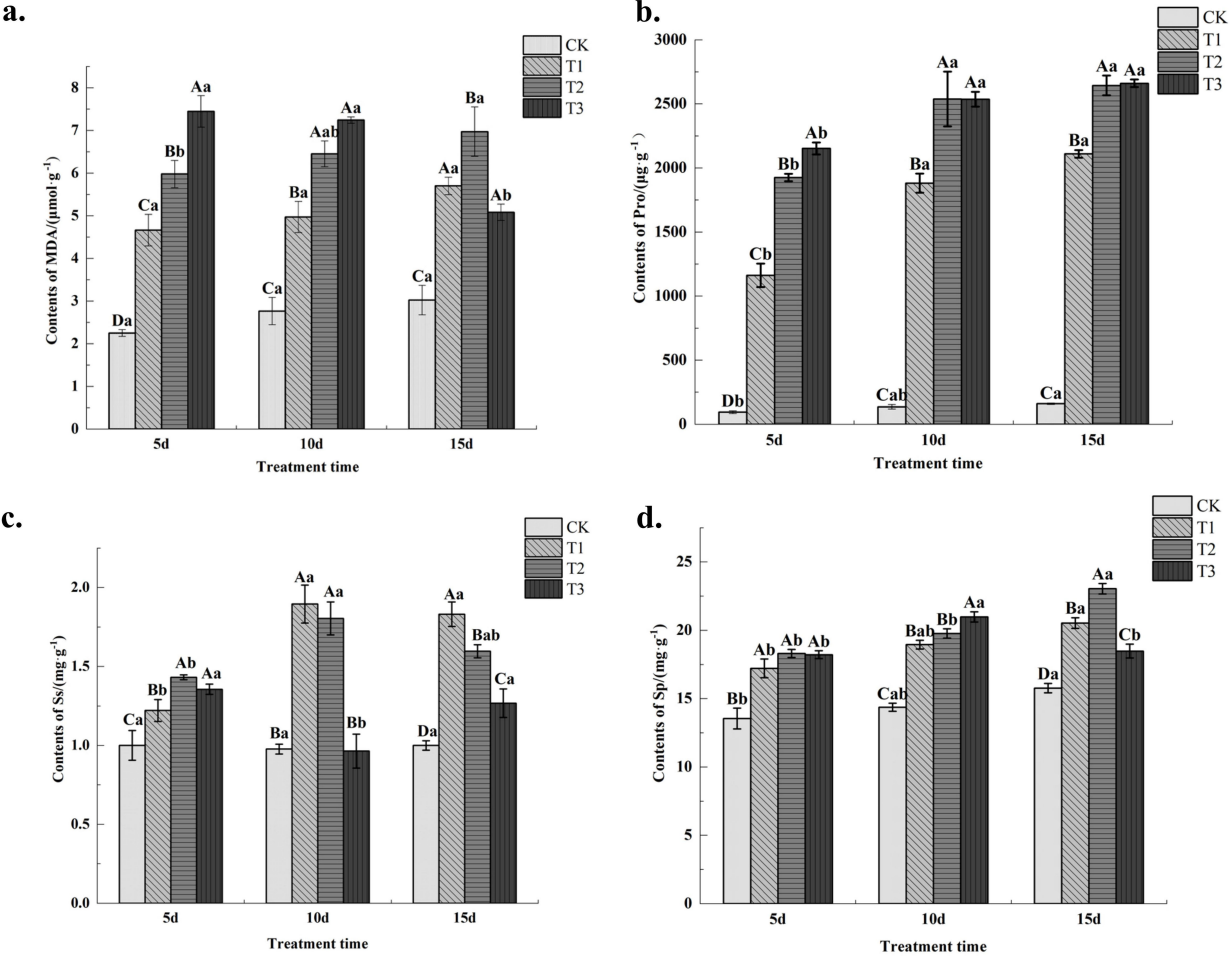
Changes of MDA, Pro, Ss and Sp content in Chamerion angustifolium seedlings leaves under drought stress. **(a)** Contents of malondialdehyde; **(b)** Contents of proline; **(c)** Contents of soluble sugar; **(d)** Contents of soluble protein .

Measurements of malondialdehyde (MDA) content revealed (**Figure 4A**) that following drought stress, MDA levels in leaves of all treated *C. angustifolium* plants were significantly higher (*P*< 0.01) than in the control. At both 5 and 10days post-stress, MDA content followed the pattern T3 > T2 > T1 > CK, with highly significant differences among treatments. At 15 days post-stress, MDA levels in the T2 treatment were significantly higher than those in the control and other treatments. As stress duration increased, MDA content in both T1 and T2 treatments showed an upward trend, while MDA content in the T3 treatment exhibited a downward trend, indicating progressive membrane damage. In contrast, under severe stress (T3), MDA content peaked at day 10 and then decreased by day 15, a pattern that may reflect severe cellular damage or tissue necrosis under prolonged extreme stress. Results indicate that MDA content in *C. angustifolium* leaf tissue increases under drought stress, with higher stress levels correlating to greater MDA accumulation. Under mild and moderate stress, MDA content progressively increased over time, whereas under severe stress, MDA content gradually decreased.

As shown in **Figure 4B**, the proline (Pro) content in all treatments was significantly higher (*P*< 0.01) than the control at different stress durations. Among the treatments, T3 > T2 > T1, with highly significant differences between treatments (*P*< 0.01). Proline content within the same drought treatment increased with prolonged stress duration. Results indicate that higher drought stress levels correlate with elevated proline content, and prolonged stress duration leads to greater proline accumulation.

Measurements of soluble sugar (Ss) content revealed (**Figure 4C**) that soluble sugar levels in all treatments were significantly (*P*< 0.05) or extremely significantly (*P*< 0.01) higher than the control level at different stress durations. At 5 days of stress, soluble sugar content was highest in the T2 treatment. At 10 and 15 days of stress, soluble sugar content followed the pattern T1 > T2 > T3, with highly significant differences among treatments. As stress duration increased, soluble sugar content in the leaves of *C. angustifolium* seedlings under T1 and T2 treatments first increased and then decreased, while T3 treatment showed a pattern of first decreasing and then increasing. Results indicate that all drought stress treatments promoted soluble sugar accumulation in *C. angustifolium* seedlings. Among these, the T2 treatment exhibited the highest soluble sugar accumulation under short-term stress, while the T1 treatment showed the highest accumulation under both medium-term and long-term stress.

Under drought stress, soluble protein (Sp) content in leaves of all treated *C. angustifolium* seedlings was significantly higher (*P*< 0.01) than in the control (**Figure 4D**). At 5 days post-stress, soluble protein content followed the pattern T2 > T3 > T1, with no significant differences among treatments. At 10 days post-stress, soluble protein content showed T3 > T2 > T1, with significant (*P*< 0.05) or extremely significant (*P*< 0.01) differences among treatments, except T2 was higher than T1 but not significantly so (*P* > 0.05). At 15 days of stress, soluble protein content showed T2 > T1 > T3, with highly significant differences among treatments. As stress duration increased, soluble protein content in T1 and T2-treated *C. angustifolium* seedlings exhibited an upward trend, while T3-treated seedlings showed an initial increase followed by a decrease. Results indicate that drought stress at various intensities promoted soluble protein accumulation. Among these, T3 treatment exhibited the highest soluble protein accumulation under short-term and medium-term stress, while T2 treatment showed the highest accumulation under long-term stress.

### 3.6 Effects of drought stress on antioxidant enzyme activity in young leaves of *C. angustifolium*

**Figure 5:**
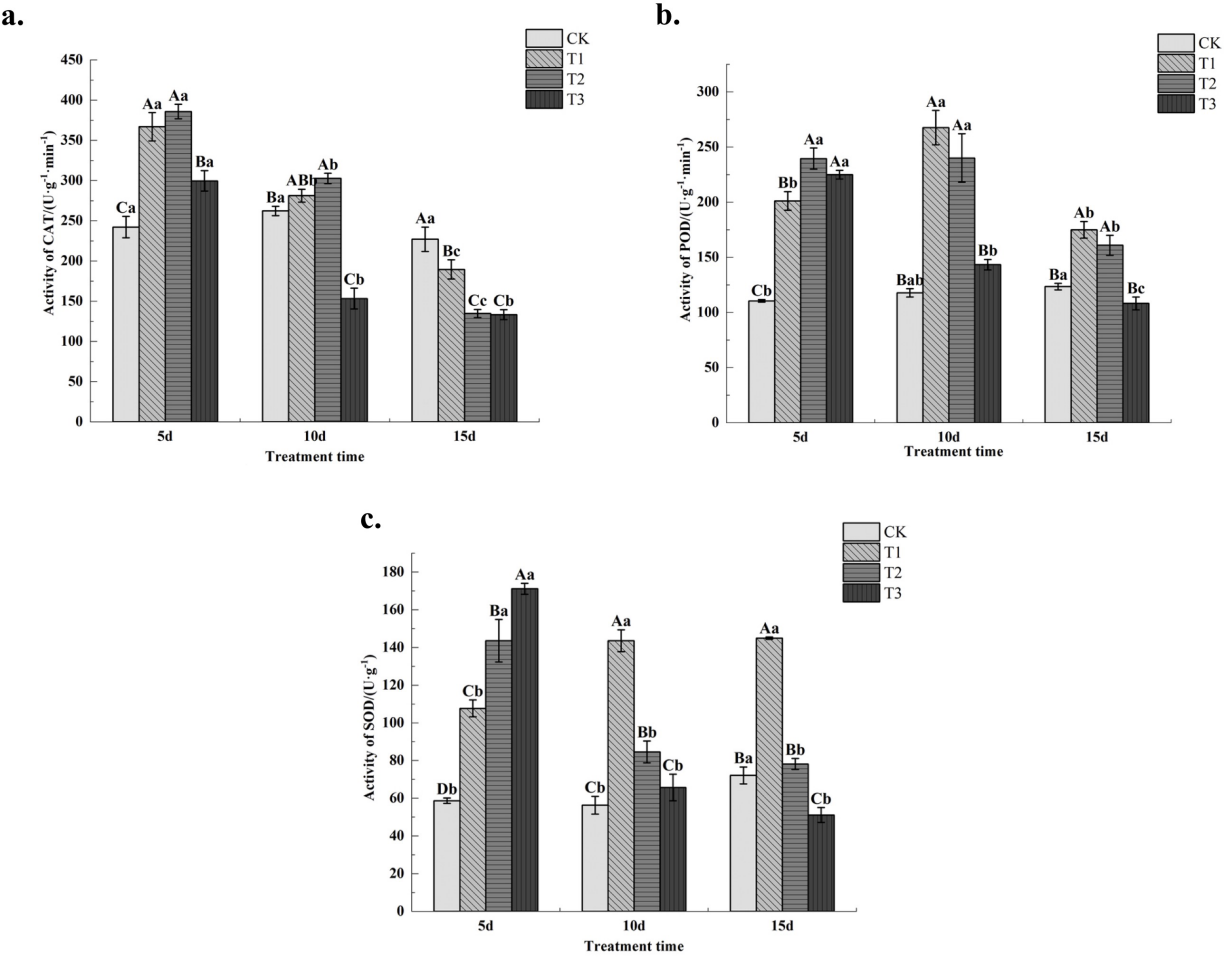
Changes of CAT, POD, SOD content in Chamerion angustifolium seedlings leaves under drought stress. **(a)** Activity of catalase; **(b)** Activity of peroxidase; **(c)** Activity of superoxide dismutase.

Measurements of CAT (catalase) activity revealed (**Figure 5A**) that the T1 treatment exhibited extremely significant (*P*< 0.01) higher activity than the control and T3 treatment at 5 days post-stress. At 10 days post-stress, it was higher than the control but the difference was not significant (P > 0.05), while it was extremely significantly higher than the T3 treatment. At 15 days post-stress, it was significantly lower than the control but extremely significantly higher than the other two treatments. CAT activity in the T2 treatment was higher than the control and other treatments at both 5 and 10 days of stress, showing highly significant differences from the control and T3 treatment but no significant difference from the T1 treatment (*P* > 0.05). At 15 days of stress, it was highly significantly lower than the control and T1 treatment but slightly higher than the T3 treatment. CAT activity in the T3 treatment was significantly higher than the control at 5 days of stress, but showed the lowest levels at both 10 and 15 days of stress, being extremely significantly lower than the control. CAT activity decreased with prolonged stress duration under the same drought treatment. Results indicate that CAT activity was highest under the T2 treatment during short-term stress, with all treatments showing enhanced CAT activity. During medium-term stress, CAT activity decreased under the T3 treatment, while all three treatments exhibited inhibited CAT activity during long-term stress.

Measurements of POD (peroxidase) activity revealed (**Figure 5B**) that POD activity in the T1 treatment was extremely significantly (*P*< 0.01) higher than the control at all stress time points. At 10 and 15 days of stress, it was extremely significantly higher than the T3 treatment and higher than the T2 treatment, though the difference was not significant (*P* > 0.05). POD activity in the T2 treatment was significantly higher than the control at all stress time points *(P*< 0.01). At 5 days of stress, it was significantly higher than the T1 treatment and higher than the T3 treatment, though the difference was not significant. POD activity in the T3 treatment was significantly higher than the control at 5 days of stress. At 10 and 15 days of stress, it showed no significant difference from the control but was significantly lower than the other two treatments. As stress duration increased, POD activity in T1-treated *C. angustifolium* seedlings first rose then declined, while POD activity in T2 and T3 treatments showed a consistent downward trend over time. Results indicate that POD activity was enhanced under both short-term and medium-term drought stress treatments, whereas it was suppressed under long-term drought stress in the T3 treatment.

Measurements of superoxide dismutase (SOD) activity revealed (**Figure 5C**) that SOD activity was higher in all treatments than in the control, except for the T3 treatment at 15 days of stress, which was significantly lower than the control. At 5 days of stress, SOD activity followed the pattern T3 > T2 > T1, with extremely significant differences among treatments (*P*< 0.01). At 10 and 15 days of stress, SOD activity showed T1 > T2 > T3, with significant (*P*< 0.05) or extremely significant (*P*< 0.01) differences among treatments. As stress duration increased, SOD activity in T1-treated *C. angustifolium* seedlings showed an upward trend, while SOD activity in T2 and T3 treatments exhibited a downward trend with prolonged stress. Results indicate that under short-term stress, SOD activity was enhanced in all three treatments, with the highest activity observed in the T3 treatment. Under medium-term stress, SOD activity peaked in the T1 treatment, whereas under long-term stress, SOD activity in the T3 treatment was suppressed.

### 3.7 Comprehensive analysis and evaluation of drought tolerance of *C. angustifolium* Seedlings

#### 3.7.1 Correlation analysis

**Figure 6:**
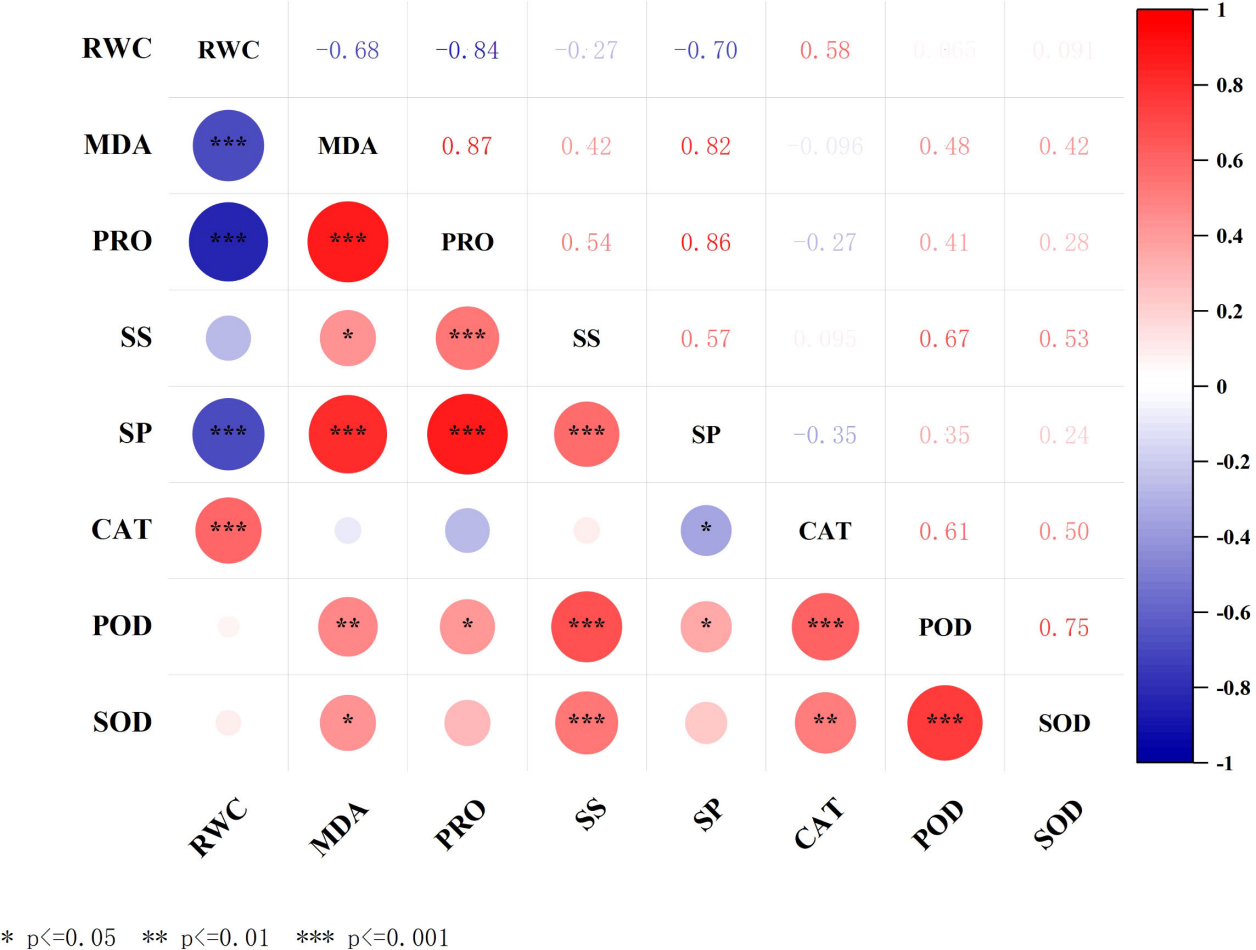
The correlation heat map. RWC, relative water content; MDA, malonaldehyde; PRO, proline; SS, soluble sugar; SP, soluble protein; CAT, catalase; POD, peroxidase; SOD, superoxide dismutase.

Correlation analysis revealed (**Figure 6**) varying degrees of correlation among morphological and physiological indicators in *C. angustifolium*. The plant’s relative water content (RWC) exhibited extremely significant negative correlations with malondialdehyde (MDA) and superoxide dismutase (SOD) (*P*< 0.01), and extremely significant positive correlations with catalase (CAT) (*P*< 0.01). MDA showed extremely significant positive correlations with both PRO and SP (*P*< 0.01), extremely significant positive correlation with POD (*P*< 0.01), and significant positive correlations with SS and SOD (*P*< 0.05); PRO of *C. angustifolium* showed a significant positive correlation with POD *(P*< 0.05); SS exhibited a highly significant positive correlation with both POD and SOD (*P*< 0.01); SP showed a significant positive correlation with POD (*P*< 0.05); and CAT demonstrated a highly significant positive correlation with both POD and SOD (*P*< 0.01).

#### 3.7.2 Principal component analysis

**Figure 7:**
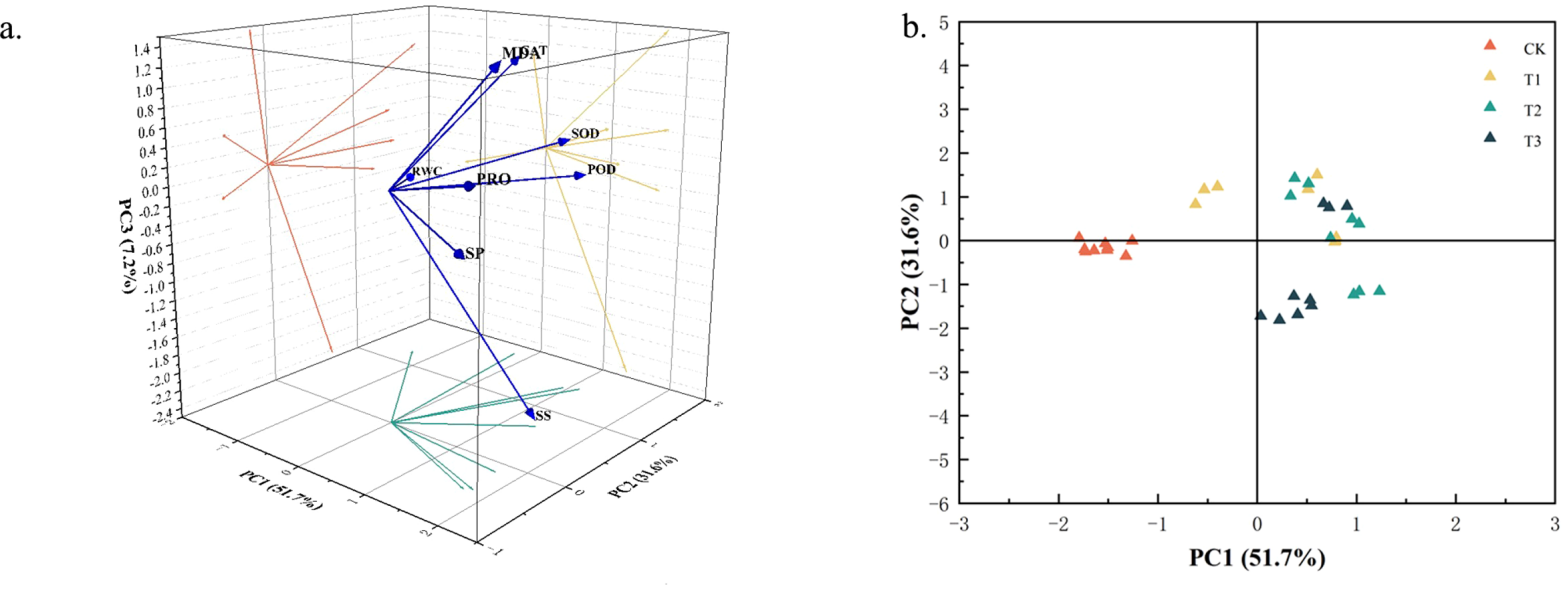
Principal component score map of physiological indexes of Chamerion angustifolium seedlings. **(a)** The first two principal component loads of indexes; **(b)** Scatter plots of samples based on PC1 and PC2.

Based on the aforementioned correlation analysis results, this study further employed principal component analysis (PCA) to reduce the dimensionality of the data (**Figure 7**). Two principal components with cumulative variance contribution rates exceeding 80% and eigenvalues greater than 1 were selected (**Table 3**), accounting for a cumulative contribution rate of 83.288%. The corresponding larger eigenvectors were PRO and CAT, indicating that these two indicators played a dominant role in distinguishing the different drought stress treatments in the present study (**Figure 7A**).

**Table 3:** Principal component analysis results of the physiological response of Chamerion angustifolium growth under drought stress.

Index	The first principal component	The second principal component
RWC	-0.347	0.394
MDA	0.446	-0.043
PRO	0.464	-0.142
SS	0.350	0.213
SP	0.442	-0.154
CAT	-0.060	0.566
POD	0.292	0.474
SOD	0.244	0.456
Eigen value	4.139	2.524
Variance contribution/%	51.733	31.554
Cumulative contribution rate/%	51.733	83.288

Scatter plots constructed based on PC1 and PC2 reveal the relationships among various indicators of *C. angustifolium*. As shown in **Figure 7B**, varying degrees of drought stress exert significant physiological effects on *C. angustifolium* growth. Simultaneously, the distribution range of sample plants on PC1 is greater than that on PC2. Samples from treatments T1 and T2 exhibit relatively concentrated distribution in the scatter plot, suggesting more similar physiological responses under these conditions, while those from T3 and other treatments showed more dispersed patterns, indicating greater variability in stress responses.

#### 3.7.3 Two-factor analysis

The results of the two-way ANOVA (**Table 4**) indicate that the severity of drought stress, the duration of stress, and the interaction between time and drought stress all exerted extremely significant effects (*P*< 0.01) on the relative water content, MDA content, osmotic regulator content, and antioxidant enzyme activity in the leaves of *C. angustifolium* seedlings.

**Table 4:** Two-way ANOVA analysis of effects of drought stress on physiological index of *Chamerion angustifolium* seedlings.

Index	Time	Time	Drought	Drough	Time×drought	Time×drought
	SS	F value	SS	F value	SS	F value
Relative water content	0.364	3877.939**	0.244	2592.369**	0.065	229.521**
Malonaldehyde	62.013	107.744**	72.117	125.300**	31.798	18.416**
Proline	24341995.3	576.404**	23311167.3	551.994**	8636892.74	68.172**
Soluble sugar	1.734	40.522**	2.219	51.842**	1.672	13.019**
Soluble protein	192.630	66.364**	116.002	39.965**	95.141	10.926**
Catalase	140550.806	83.512**	28495.481	16.931**	69880.702	13.841**
Peroxidase	62065.043	93.153**	45110.704	67.706**	41664.018	20.844**
Superoxide dismutase	21700.598	99.761**	16408.798	75.434**	33435.632	51.236**

SS is sum of squares, **, Extremely significant correlation (*P* < 0.01).

## 4 Discussion

### 4.1 The effect of PEG-6000 solution simulated drought stress on the germination of *Chamerion angustifolium* seeds

PEG-6000 solutions effectively simulate soil water deficits by generating controlled osmotic stress. When facing drought stress, seeds serve as the starting point of life, and seed germination indicators are crucial data reflecting their drought tolerance (Du et al., 2022). Different concentrations of PEG-6000 stress significantly influenced the germination of *C. angustifolium* seed, consistent with study on *Triticale hexaploide* seed germination Guo et al. (2022). Moderate drought stress, particularly at 4% PEG-6000, effectively enhanced both germination rates and physiological activity of *C. angustifolium* seeds, with germination parameters significantly higher than at 2% (**Table 2**), suggesting a hormetic effect where mild osmotic stress stimulates germination. When osmotic stress intensity exceeded this critical concentration, all germination parameters exhibited a decline inversely proportional to PEG-6000 concentration. Under high osmotic pressure conditions (12%), germination parameters reached their experimental minimum values, indicating that excessive osmotic stress damages seed physiological functions, thereby significantly inhibiting the germination process. This result reveals a nonlinear relationship between drought stress intensity and seed germination performance, consistent with findings by Wang et al. (2023). Relative drought damage rate reflects the extent of drought injury during seed germination and serves as an indicator of seed drought tolerance during the germination phase. At the 12% concentration, the relative drought damage rate reached 100%, indicating that seeds cannot germinate or grow normally under this stress level once the concentration exceeds 12%. This may occur because *C. angustifolium* seeds rapidly absorb water required for germination during the initial stage, and excessively high osmotic pressure severely inhibits seed swelling, thereby preventing germination (Batool et al., 2022). Thus, it is evident that *C. angustifolium* seeds exhibit a certain degree of drought tolerance, with seeds capable of tolerating mild to moderate osmotic stress (0–8% PEG). This finding aligns with the germination characteristics of *Triticum aestivum* seeds studied by Mahpara et al. (2022), where increasing PEG-6000 solution concentration reduces seed osmotic regulation function, impedes water absorption, and disrupts antioxidant enzyme structures, ultimately leading to decreased seed germination rates.

The growth rate and fresh weight of the radicle and plumule serve as crucial indicators for assessing seed germination, most effectively reflecting the growth and development status of plant seeds during the germination phase (Lu et al., 2022). Among these, the radicle primarily influences seed germination by affecting root water uptake. Research indicates that the rate of root formation and the extent of radicle elongation exhibit significant correlations with the intensity of water stress (Gholami et al., 2022). This experiment demonstrated that the growth of both the radicle and plumule was significantly reduced in all drought stress treatments compared to the CK, indicating that the radicle and plumule of *C. angustifolium* are highly sensitive to drought stress. This finding is consistent with the results of on *liquorice* seedlings Hou and Ma (2022).

Soluble sugars maintain cellular structural stability by regulating osmotic potential, thereby ensuring the normal progression of various physiological metabolic activities. The accumulation levels of sugar metabolites exhibit a significant positive correlation with plant stress resistance, and this protective mechanism plays a crucial role in sustaining fundamental life functions under stress conditions (Tounekti et al., 2020). Data on the effects of PEG-6000 on osmotic regulator content in *C. angustifolium* seeds indicate that soluble sugar content in seeds increases with intensifying drought stress. This phenomenon may be associated with enhanced amylase activity, accelerating the conversion of stored starch into soluble sugars. Concurrently, the pattern of soluble protein content changes indicates that water stress significantly impacts the conversion of nitrogen metabolites within seeds, consistent with findings by Zhang et al. (2024). The substantial increase in soluble protein content reveals that *C. angustifolium* seeds activate an intrinsic stress response system during germination. Under PEG-6000 stress, soluble protein content first increased and then decreased. This pattern likely reflects that during mild or moderate drought, plants increase soluble proteins to maintain cellular osmotic balance, whereas severe drought exceeds their self-regulatory capacity, leading to protein degradation. This finding is consistent with the results reported by Kalantar Ahmadi and Eyni-Nargeseh (2023). Collectively, experimental data indicate that *C. angustifolium* seed possesses a certain degree of drought resistance.

### 4.2 The effects of drought stress on the physiological characteristics of *C. angustifolium* seedlings

Drought stress disrupts cellular water balance, membrane integrity, and metabolic processes, triggering antioxidant defense systems to scavenge reactive oxygen species and mitigate oxidative damage (Zafar et al., 2023; Chieb and Gachomo, 2023; Nyaupane et al., 2024).

Relative water content comprehensively reflects the water balance status and regulatory capacity within plants (Kuromori et al., 2022). In this study, the relative water content of *C. angustifolium* leaves exhibited a decreasing trend under varying stress intensities and durations. This indicates that as drought stress intensified and stress duration prolonged, water deficit within the seedlings increased while their water retention capacity diminished. These findings align with the results reported by on *Brassica napus* seedlings Batool et al. (2023).

Malondialdehyde (MDA) is a biomarker of lipid peroxidation, reflecting oxidative damage to cell membranes under stress (Yang et al., 2021). In this study, MDA content in C. angustifolium leaves increased significantly under all drought treatments compared to the control, consistent with previous reports (González, 2023; Bogati and Walczak, 2022). However, an interesting trend was observed under severe stress (T3): MDA content increased from day 5 to day 10, but decreased by day 15 (**Figure 4a**). While this may seem counterintuitive, such a decline under prolonged severe stress can be explained by several mechanisms. First, prolonged extreme stress can lead to tissue necrosis and cell death; when cells die, lipid peroxidation may cease, resulting in reduced measurable MDA in surviving or sampled tissues (Mittler, 2002). Second, the decline in MDA coincided with a decrease in antioxidant enzyme activities (CAT, POD, SOD) under T3 at day 15 (**Figure 5**), indicating a collapse of the protective system that may disrupt MDA generation (Xu et al., 2021). Third, plants possess mechanisms to metabolize MDA, which might become significant under chronic stress (Yamauchi et al., 2008). Importantly, this decline does not indicate recovery, but rather reflects severe physiological deterioration, including cell death and collapse of antioxidant defense, consistent with the observed growth inhibition under the same treatment.

Proline, soluble sugars, and soluble proteins enhance water absorption capacity by reducing cellular water potential, thereby ensuring the normal progression of fundamental physiological metabolism. Hassan et al. observed increases in proline and soluble sugar content Hassan et al. (2023). In this study, under mild stress, medium-duration stress, and long-duration stress, soluble sugar and soluble protein contents were significantly higher than the control, indicating that leaves accumulate large amounts of soluble sugars and proteins to counteract drought stress damage to *C. angustifolium*. Similar findings were reported in physiological and biochemical studies of soybean under soil drought stress (Poudel et al., 2023). Following drought stress, soluble sugar content in the tissues of leaves of *C. angustifolium* gradually increased but declined with prolonged stress duration and intensified drought severity. This decrease may result from reduced chlorophyll content and diminished photosynthetic products, leading to lower soluble sugar accumulation and restricted soluble protein synthesis (Geng et al., 2024). Numerous studies indicate that elevated free proline levels under drought stress represent an adaptive response to stress (Abreha et al., 2022). Under all three drought treatments in this study, proline content significantly increased, reaching 15.6 times that of the control after 15 days of severe drought stress. This indicates that severe stress conditions significantly induce proline production.

Superoxide dismutase, peroxidase, and catalase are key protective enzymes. Under drought stress conditions, plant cells produce excessive reactive oxygen species. Sustained oxidative stress disrupts the function of the biomembrane system, ultimately leading to programmed cell death. To counter this threat, superoxide dismutase and peroxidase act synergistically to maintain the integrity and functionality of cell membrane structures (Hura et al., 2022). Pantha et al. studied the desert plant *Triticum* genus and found that antioxidant enzyme activity increased under mild and moderate drought stress to scavenge excess ROS Pantha et al. (2024). In this experiment, antioxidant enzyme activity showed an upward trend under mild stress, while under moderate and severe stress, it first increased and then decreased. This indicates that *C. angustifolium* can counteract oxidative damage by upregulating antioxidant enzyme activity in response to mild stress. However, when drought stress intensifies, the catalytic activity of these key antioxidant enzymes declines, suggesting that accumulated ROS exceed the plant’s regulatory capacity—a finding consistent with the results reported by Hu et al. (2023).

Correlation analysis results indicate that the relative water content of *C. angustifolium* seedlings exhibits significant correlations with multiple physiological indicators. Specifically, tissue water content shows a negative correlation trend with plant growth rate, malondialdehyde concentration, and proline concentration. This phenomenon indicates that as drought stress intensity increases, the water status within plant cells progressively deteriorates, leading to significant changes in osmotic potential. To counteract these changes, the accumulation of osmotic regulatory substances increases. This response mechanism reflects an important strategy by which plants adapt to environmental stress through metabolic regulation.

These findings were further validated and refined through principal component analysis (PCA). The PCA identified proline (Pro) and catalase (CAT) as the two physiological indicators that most strongly reflected the drought stress responses observed in this study (**Figure 7**, **Table 3**).This result is physiologically coherent: proline serves as a key osmoprotectant that accumulates under water deficit to maintain cellular turgor and protect macromolecules, while CAT is a central enzyme in the antioxidant system that scavenges hydrogen peroxide and mitigates oxidative stress.

Under mild or short-term drought, the coordinated upregulation of these components helps to sustain physiological function. However, as drought intensity or duration increases—surpassing the plant’s compensatory capacity—the sustained elevation of proline and the dynamic response of CAT activity collectively reflect the degree of osmotic adjustment and oxidative management. Thus, proline accumulation and CAT activity emerge as key physiological traits associated with the adaptive responses of *C. angustifolium* to drought under the conditions tested.

## 5 Conclusion

This study analyzed seed germination under drought stress, seed osmotic regulators, seedling osmotic regulators, membrane system stability, and antioxidant enzyme systems in *Chamerion angustifolium*. Results indicate that mild stress promotes seed germination, seedling growth, osmotic regulation capacity, and antioxidant enzyme activity. As stress intensity increases and duration prolongs, seed germination and seedling antioxidant enzyme activity are inhibited, while seedling malondialdehyde and proline levels increased significantly, and soluble sugar and soluble protein contents decreased. Thus, *C. angustifolium* possesses a degree of drought tolerance. Mild stress promotes seed germination, and seedlings can adapt to short-term stress and mild drought (soil moisture content 70% ± 5%) by increasing osmotic regulator content and enhancing antioxidant enzyme system activity. However, under prolonged and severe drought stress (soil moisture content 30% ± 5%), the plant exceeds its self-regulatory capacity. Correlation analysis indicates that under drought stress, various indicators interact to counteract the stress, thereby maintaining normal physiological functions. Principal component analysis further highlighted that proline and catalase were the primary physiological traits associated with the drought stress responses observed in this study.

## Data Availability

The original contributions presented in the study are included in the article/supplementary material. Further inquiries can be directed to the corresponding author.
